# Skeletal muscle tissue engineering: strategies for volumetric constructs

**DOI:** 10.3389/fphys.2014.00362

**Published:** 2014-09-22

**Authors:** Giorgio Cittadella Vigodarzere, Sara Mantero

**Affiliations:** Department of Chemistry, Materials and Chemical Engineering “Giulio Natta”, Politecnico di MilanoMilano, Italy

**Keywords:** skeletal muscle, tissue engineering, physical stimulation, mechanobiology, vascularization

## Abstract

Skeletal muscle tissue is characterized by high metabolic requirements, defined structure and high regenerative potential. As such, it constitutes an appealing platform for tissue engineering to address volumetric defects, as proven by recent works in this field. Several issues common to all engineered constructs constrain the variety of tissues that can be realized *in vitro*, principal among them the lack of a vascular system and the absence of reliable cell sources; as it is, the only successful tissue engineering constructs are not characterized by active function, present limited cellular survival at implantation and possess low metabolic requirements. Recently, functionally competent constructs have been engineered, with vascular structures supporting their metabolic requirements. In addition to the use of biochemical cues, physical means, mechanical stimulation and the application of electric tension have proven effective in stimulating the differentiation of cells and the maturation of the constructs; while the use of co-cultures provided fine control of cellular developments through paracrine activity. This review will provide a brief analysis of some of the most promising improvements in the field, with particular attention to the techniques that could prove easily transferable to other branches of tissue engineering.

## Introduction

Skeletal muscle tissue represents the most abundant tissue type in the human body, amounting to 60% of the average weight. It is a metabolically active tissues requiring a constant flow of nutrients and metabolites, provided by an extensive capillary network forming an organized branching pattern throughout the fibers (Dennis and Kosnik, 2000; Liu et al., 2012).

Skeletal muscle tissue engineering (SMTE) aims to replicate the structure and function of skeletal muscle tissue *in vitro* and *in vivo*, to obtain valid models and functional constructs whose ultimate goal is the implantation as a therapeutic device (Ostrovidov et al., 2014). This discipline presents unique challenges compared to other tissue engineering strategies that have shown promise in clinical applications (Naito et al., 2003; Atala et al., 2006; Macchiarini et al., 2008; McAllister et al., 2009), in that small lesions and defects in skeletal muscle tissue seldom, if at all, require chirurgical intervention and transplantation; injuries heal spontaneously through an inflammation related mechanism (Ciciliot and Schiaffino, 2010; Turner and Badylak, 2012) involving resident stem cells, named satellite cells (SCs) (Pannérec et al., 2012).

Lesions so extensive that the function of the muscle is impaired are commonly designated volumetric muscle losses (VMLs) (Grogan et al., 2011); these lesions, mainly due to traumatic injury and chirurgical resection of tumors, are treated using free standing flaps as gold standard, a procedure that is inevitably associated with morbidity at the donor site and limited successful outcomes (Agostini et al., 2013). The use of flaps, rather than grafts, is a necessity because transplants of this kind cannot survive without an independent blood supply Similarly, clinically relevant SMTE constructs would require autonomous vascular networks to avoid central (Griffith et al., 2005; Bae et al., 2012; Shandalov et al., 2014).

However, in a clinical setting the goal of an SMTE device is the reestablishment of function rather than the original homeostasis of the tissue, there have been efforts to engineer constructs in which the contractile ability of the muscles is restored through functional fibrotic structures (Corona et al., 2013b); as long as there is a continuity in muscle architecture that allows the force transmission even a fibrotic structure formed from and acellular scaffold will ameliorate VML (Aärimaa et al., 2004).

Given the unique requirements in the treatment of the lesions in musculoskeletal tissues, SMTE constructs are designed to ameliorate large tissue defects and can only have limited clinical application without a vascular system to support the cellular component; similar considerations must be undertaken in the case of preclinical constructs, as large three dimensional structures have proven to replicate more closely the physiology of the original tissues (Rouwkema et al., 2011). It is necessary to mention that lesions occurring in craniofacial skeletal muscles (Garland and Pomerantz, 2012) must be set apart from the previous generalizations, as these tissues present a different cellular milieu compared to the muscles of the limbs and trunk (Kelly, 2010; Lemos et al., 2012); as such, it could be argued that different models should be produced to study these pathologies, though discipline may still prove of use for clinically applicable constructs due to the relatively restricted size of the lesions and the difficult application of flaps in this region.

### Skeletal muscle structure

The structure of skeletal muscle is inherently correlated to its function; this tissue is characterized by a highly ordered structure composed of parallel elements that can be summarily divided in myofibrils, muscle fibers and fascicles. A myofibril is the cytoplasmic molecular machinery capable of actuating muscle contraction through the relative movement of two interlocking macrostructures, the thin actin filaments and thick myosin filaments (Huxley and Hanson, 1954). Myofibrils are bundled within the massive cytoplasm of a multinucleated syncytium, the muscle cell or myofiber; motoneuron connections in the cell membrane (the sarcolemma) regulate the flow of calcium ions through the sarcoplasmic reticulum, necessary for the contraction of the myofibrils.

Myofibers constitute the parenchyma of skeletal muscle, and are bundled in a complex ECM structure which connects them to the muscle-tendon junction through three fibrous layers: the endomysium, surrounding individual myofibers, the perimysium, found over fascicles and the epimysium which cover the entire muscle.

**Figure 1:**
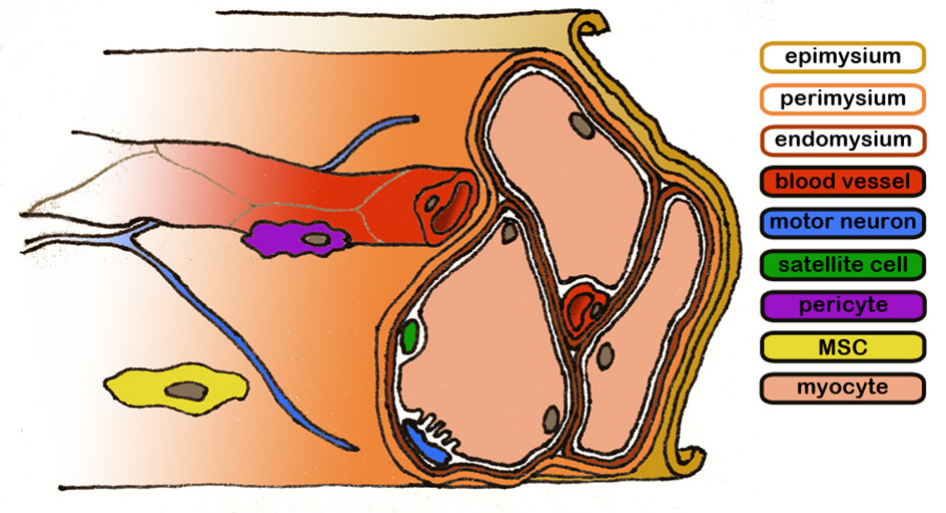
**Representative diagram of the skeletal muscle structure**. Connective layers and cell populations of particular interest are evidenced. The biological components are not drawn to scale.

The fine structure is particularly evident in decellularized muscles, where the bare extracellular matrix (ECM) forms structures not unlike bundles of flexible straws (Lieber and Fridén, 2000), which is the principal component of the muscle's anisotropic response to stress (Takaza et al., 2013). Blood vessels run along the fascicles in the perimysium and penetrate into the endomysium, forming capillaries that project around the myofibers; due to the elevated metabolic need of the skeletal muscle tissue, each myofiber is connected to a capillary (Figure 1). At the same time, the axons of motor neurons, which transmit the synaptic signal to the myofibers. Following the route traced by the blood vessels, motor neurons terminate into neuromuscular junctions in the proximity of sarcolemma-SR connections (triads); the association between the vascular and the nervous system is thought to start from the developmental phase of the organism (Carmeliet and Tessier-Lavigne, 2005) and is conserved in regenerative processes involving a common mesenchymal progenitor cell able to generate musculoskeletal, nervous and vascular tissues (Tamaki et al., 2013).

The cellular machinery involved in the maintenance and regeneration of skeletal muscle tissue is the same responsible for. the delicate balance between hypertrophy and catabolism; these processes has been studied in detail (Joe et al., 2010; Ten Broek et al., 2010; Dellavalle et al., 2011; Turner and Badylak, 2012; Saclier et al., 2013), and three cellular populations have proven especially relevant to the regeneration process. SCs (Mauro, 1961), located between the basal lamina and the sarcolemma, are the tissue resident progenitor cells responsible for the maintenance and the continual production on myoblasts. Once activated, SCs will reveal heterogeneity in their population and will start dividing and producing muscle progenitor cells (MPCs) that eventually will become myoblasts and fuse to the existing myofibers (Boldrin et al., 2010); pathological conditions such as Duchenne muscular dystrophy (DMD) are known to deplete the reservoir of available SCs, leading to fibrosis and adipose tissue inclusion in muscular tissues.

SC pools can be replenished by muscular tissue pericytes (MPs), a population of cells found in close proximity to blood vessels and difficult to identify, as they bear no unique CD set or biochemical markers' (Dellavalle et al., 2011). These cells are capable of directing the behavior of endothelial cells (ECs), acting as guides during the formation of new blood vessels (Fuoco et al., 2014). Here, these cells have been shown to lose in part their ability to differentiate into myogenic lineage and to induce angiogenesis in nearby ECs as their host ages; nevertheless, the “myogenic/vasculogenic ability” appears to be restored after the cells are seeded on a polyethylene glycol-fibrinogen (PEG-F) surface. The mechanism underlying this behavior is yet to be elucidated, although a comparison with previous experiments by Discher et al. (2005) and Engler et al. (2006), might suggest a sensitivity to the mechanical stimuli of cellular environment.

Finally, fibro/adipogenic progenitors (FAPs) are a PDGFRα^+^ myogenic cell population, found in state of quiescence near blood vessels, which activates in case of injury to the muscle (Joe et al., 2010). FAP cells are known to promote the differentiation of MPCs but do not have any capability to generate muscle tissue on their own; on the contrary, they appear to be the main source of the fibrotic and adipose tissues found in pathological muscles (Uezumi et al., 2011). As such, these cells have been studied as a target for with microRNA (miRNA) treatments intended to reduce the symptoms of the DMD syndrome through the upregulation of the FAP cells myogenic programming, inducing a “compensatory regeneration” (Mozzetta et al., 2013; Saccone et al., 2014).

The interplay between these cell populations is fundamental for the continuous renewal of skeletal muscle tissue, required to compensate the damage occurring in day-to-day activities (Järvinen et al., 2013); however, pathological states also require the activation of the same cells in different processes that can result in the regeneration of the tissue or the formation of a fibrotic scar.

### Skeletal muscle repair process

The regenerative process of muscle tissue is complex, and not yet understood in its entirety; different cell populations interact with each other and their environment to progress into outcomes which span from the reestablishment of function to fibrosis to pathological states of chronic inflammation (Koopman et al., 2014). A brief recapitulation of the phases of this process and the cellular populations involved will provide a background for the *in vivo* implantation of SMTE constructs, and will define some of the challenges involved in the development of *in vitro* devices. Skeletal muscle regeneration can be summarily divided into three overlapping phases: inflammation and activation of stem cells, differentiation and deposition of a provisional ECM and finally maturation of the tissues and remodeling of the ECM (Ciciliot and Schiaffino, 2010).

In the first phase (the peak is reached in 2 h) of the regeneration process neutrophil granulocytes, innate immune response cells with antimicrobial activity, enter the lesion from the blood stream; their function is disputed but it has been proven that they possess antimicrobial activity, exert phagocytosis on tissue debris (Teixeira et al., 2003) and that they can stimulate vascularization through production of VEGF-A (Christoffersson et al., 2012); at the same time, however, this cell population releases cytotoxic compounds that can interfere with the regeneration process (Tidball and Villalta, 2010).

Another population of immune cells, the eosinophils, invade the tissue in this phase and activate FAP cells through the release of IL-4 (Heredia et al., 2013); after this initial stage the macrophages enter the lesion site: pro-inflammatory CD68+ CD163− macrophages (M1), which reach maximal concentration 24 h after the injury, followed by a switch in polarization leading to alternative activated, CD68− CD163+ macrophages (M2) with anti-inflammatory activity (Tidball and Villalta, 2010; Mantovani et al., 2013; Tidball et al., 2014). This activity bridges the inflammatory-proliferative phase with the following one, anti-inflammatory and differentiative; the shift is driven by the interaction between the macrophages and the SCs, which activate in response to the tissue injury and start an asymmetric replication cycle to produce myogenic progenitors (MP): simplistically, it can be said that during the initial phase SC division is stimulated by M1 macrophages via TNFα, then differentiation occurs through IL-4 and IL-10 intervention by M2 cells. Whereas quiescent SC express a characteristic set of markers including Pax-7, M-Cadherin and CD 34, activated SC rapidly change this configuration as they proliferate; diminishing Pax -7 expression and producing basic helix-loop-helix (bHLH) transcription factors as they differentiate into committed MPs; these cells form multinucleated myotubes that express neonatal MyHC (Tidball et al., 2014). It is important to mention that only a fraction of the SC population possesses stemness and is capable of perpetuate itself and generate daughter cells of different phenotypes (Wang and Rudnicki, 2012).

At the same time, FAP cells rapidly multiply under IL-4 stimulation, enforcing an ambivalent role in the context of wound healing: while they provide MPs with growth factors necessary for their differentiation in myocytes, they are also the main origin of fibrosis in tissue repair and adipose tissue in pathological conditions (Uezumi et al., 2011; Heredia et al., 2013). In parallel, a population of connective tissue fibroblasts counterbalances FAPs' activity by dampening SC differentiation through the formation of a muscle connective tissue (MCT) and reciprocated paracrine signals (Murphy et al., 2011).

The final phase in muscle regeneration there is the resolution of the provisional ECM and the formation of a definitive structure that connects the stumps of the damaged fibers; in this process the basal lamina acts as a guide for the growth of the myofibers (Schmalbruch, 1976). The remodeling of the fibrotic tissue and the subsequent regeneration of the skeletal muscle, or lack thereof, is dependent on a variety of factors, including the vascularization and the innervation of the healing area. Generally, two positive outcomes can be envisioned as the resolution of the third phase: the regeneration of the muscle tissue, with the original architecture, or the formation of scar tissue and the separation of the muscle fibers (Ciciliot and Schiaffino, 2010; Turner and Badylak, 2012).

## Vascularization

Vascular supply in the muscle is provided by a highly organized network of vessels, which are for the most part aligned with the direction of the muscle fibers. The organization of the structure is highly hierarchical an provides each myofiber with blood supply; the primary arteries run along the direction of the muscle fibers, and diverge inside the muscle through the epimysium via feed arteries; those are short branches that penetrate inside the muscle at an angle, or perpendicularly. Secondary arteriolar branches run again parallel to the muscle fibers, and then arterioles, through the perimysium, and finally connect to a microvascular unit composed of arterial and venous capillaries that follows the endomysium.

SMTE constructs, as well as any other tissue engineering construct which exceeds in any dimension the diffusion limit for oxygen and nutrients (Karande et al., 2004; Griffith et al., 2005; Radisic et al., 2006), require a specialized mean of delivery of such substances and removal of metabolic byproducts if they are to maintain the viability of seeded cells. Without these structures the constructs are likely to form necrotic cores (Radisic et al., 2004), compromising the regeneration process.

An arbitrary divide can be made between the techniques that see the formation of vascular structures *in vitro* and those that are implanted and develop vessels *in vivo*: in the first case various avenues have been explored to generate patterns within the volume of the engineered material so that the definitive shape of the network will be informed beforehand, whereas in the second case there is no specific structural conformation before implantation. This discrimination is necessary as current techniques are unable to produce mature blood vessel networks *in vitro*, meaning that a maturation process will always be present in the development of such constructs, if they are to be used as implants. Constructs intended as a vascularization models are subjected instead to different degrees of maturation *in vitro*, which determine the characteristic of their vascular structures, and as such will be discussed in the first group.

It can be argued that no engineered construct can be produced that will not incur into substantial remodeling upon implantation: even an hypothetical vascularized flap undistinguishable from the host's tissue would be colonized and altered by the surrounding capillaries, meaning that vascularization of implanted devices will always comprise an *in vivo* phase. According to this reasoning, the processes described here will be grouped according to whether they focus on the production of vascular structures within the constructs or the realization of constructs capable of allowing and increasing the penetration of the host vessels upon implantation.

### *In vitro* vascularization

Approaches for *in vitro* vascularization focus on the production and maturation of structured constructs before an eventual implantation; this is realized by producing an environment favorable to the formation of vascular compartments by vasculogenic cell populations, either by mechanical placement or through directed self-assembly (Table 1).

**Table 1:** ***In vitro* models of interest**.

**References**	**Model**	**Scaffold**	**Construct type**	**Relevant details**
Salimath and García, 2014	C2C12	PEG-MAL hydrogel	*In vitro* model	3D model; variable stiffness; chemical functionalization
Egusa et al., 2013	Mouse BMMSC; C2C12	Fibrin on silicone	*In vitro* model	Differentiation due to mechanical stimulation, myogenic medium
Morimoto et al., 2013	C2C12, primary mNSC	Matrigel	*In vitro* model	Self tension, NMJ formation
Nunes et al., 2013	hPSC differentiated in cardiomyocites	Autologous ECM; collagen	*In vitro* model	Self assembly; electrical stimuli; wire structure
Palamà et al., 2013	C2C12	–	*In vitro* model	μtopography; LbL polyelectrolyte on glass
Shah et al., 2013	Human masseter muscle cells	Biodegradable bacteriostatic phosphate glass fibers; collagen	*In vitro* model	μpatterning; self contraction
Snyman et al., 2013	C2C12; pHMB	Neutralized collagen I hydrogel	*In‘vitro* model	Self contraction; reproducibility
Wang et al., 2013	GFP-C3H myoblasts	Fibrin gel	*In vitro* model	Laminin, agrin formation; formation of NM receptors
Bian et al., 2012	Neonatal sprague-dawley rat SM myoblasts	Matrigel-fibrin mesoscopically structured hydrogel	*In vitro* model	Self contraction; analysis of SM networks; electrical stimuli
Hosseini et al., 2012	C2C12	μstructured gelatin methacrylate	*In vitro* model	μpatterning; size analysis; electrical stimuli
Monge et al., 2012	C2C12	Patterned film (polyelectrolyte)	*In vitro* model	μpatterning; stiffness modification
Sharples et al., 2012	Aged C2C12 (54x)	Collagen	*In vitro* model	Self contraction; reduced contractile force
Weist et al., 2012	Primary cells from F344 soleus	Self produced ECM	*In vitro* model	TGF-β 1 effect evaluation; electrical stimuli; self contraction into 3D
Elmer et al., 2011	C2C12; C3H10T1/2	Electrospun PS	*In vitro* model	Chemical functionalization; μtopography
Li et al., 2011	Primary myoblasts from C57/B6 mice; embryonic fibroblasts from E13/CF1 mice	Self produced ECM; fibrin	*In vitro* model	Self contraction and generation of 3D structures; cocolture
Pennisi et al., 2011	C2C12	Collagen I	*In vitro* model	Mechanical conditioning, uniaxial vs. multiaxial
van der Schaft et al., 2011	H5V EC; C2C12	Collagen I; self produced ECM	*In vitro* model	Self contraction; cocolture; effect of paracrine VEGF on vascularization and alignment
Lam et al., 2009	Spreague-dawley rat soleus SC	Self-produced ECM	*In vitro* model	μpatterning; self assembly into 3D (roll-up)
Riboldi et al., 2008	C2C12	Degrapol	*In vitro* model	μpatterning
Engler et al., 2004	C2C12; primary human fibroblasts	Patterned polyacrylamide gel	*In vitro* model	Mechanoregulation; cellular substrate
Dennis et al., 2001	Primary sprague-dawley rat SC and FBs	Self produced ECM	*In vitro* model	Self contraction; self structuring 3D (roll up)

*BMMSC: Bone Marrow Mesenchimal Stem Cell. EC, Endothelial Cell; FB, FibroBlast; GFP, Green Fluorescent Protein; hPSC, human Pluripotent Stem Cell; mNSC, mouse Neural Stem Cell; NM, NeuroMuscular; NMJ, NeuroMuscular Junction; PEG-MAL, PolyEthylene Glycol-MALeimide; pHMB, primary Human MyoBlast; SC, Satellite cells; SM, Skeletal Muscle*.

Constructs belonging to the first group are arguably the most numerous: they comprise those devices which implement structural and mechanical cues to direct the growth of vascular cells, most often ECs, endothelial progenitor cells (EPCs) and vascular smooth muscle cells (VMSCs). The methods used to produce the patterns include, most notably, sacrificial three-dimensional patterns (Miller et al., 2012; Hooper et al., 2014), direct deposition of cellular suspensions (Kolesky et al., 2014), self-assembly of polyelectrolyte solutions (Leong et al., 2013) or patterned cellular sheets (Muraoka et al., 2013), and usage of decellularized scaffolds, although the best known example refers to cardiac tissue (Ott et al., 2008; Koffler et al., 2011). In particular, the cell sheet techniques recently developed (Haraguchi et al., 2012; Nagamori et al., 2013; Sakaguchi et al., 2013; Sekine et al., 2013) resulted in the production of *in vitro* vascularized tissues using culture dishes coated with poly-N-isopropylacrylamide (PNIPAM) polymer derivatives that are able to modify the cellular adhesion by changing their hydrophilicity in a temperature range compatible with cell survival (Yamato et al., 2007). Interestingly, the interposition of a collagen sheet traversed with microchannels parallel to its surface through which was flowing medium was sufficient to generate the growth of vascular structures. These results indicate that ECs are able to respond to the mechanical cues generated by shear stress in adjacent vessels, even if these vessels are simply cavities in collagen; this is in accordance with findings in the field of vascular tissue engineering (Feaver et al., 2013) which describe the result of the mechanical stimulation on the phenotype of ECs. The complex effects resulting from cyclic mechanical stimuli are a valid tool in directing the behavior of vasculogenic cells and the generation of blood vessels (Kilarski et al., 2009; Boerckel et al., 2011), but the resulting vessels lack the order and pervasiveness of the natural vasculature; a better understanding of the cellular response to mechanical stress is therefore necessary to obtain mature, lifelike constructs.

On the other hand, devices that stimulate vascular growth *in vitro* without offering a predetermined pattern are not as common since scaffolds permitting unimpeded development of vascular structures are typically hydrogels, whose plastic qualities and wide range of possible biochemical and structural modifications make them more adapt for *in vivo* settings. Nonetheless, similar scaffold have been used to obtain networks either from preexistent structures (angiogenesis) (Chiu et al., 2012), or from isolated precursors (vasculogenesis) (Raghavan et al., 2010), demonstrating a better performance upon implantation compared to non-prevascularized constructs. This is also true for non-woven polymeric scaffolds, where the random structure of the scaffold limits the control over the growth of vessels (Levenberg et al., 2005); these devices have also profited from the application of biologically-derived hydrogel coatings, which provided a more permissive to vascular growth (Lesman et al., 2011; Sadr et al., 2012). Finally, “spontaneous” *in vitro* vascularization is fundamental in engineering “myooids” (Dennis and Kosnik, 2000), self-aggregated constructs based on the mixed muscle cell population. These constructs are based on provisional ECM scaffolds synthesized by the fibroblast population present in the extract from minced muscle, and present a self-organizing vascular layout derived from the EC and the SMC population (Carosio et al., 2013).

Construct which do not present geometrically defined cues for the vascular development such as these rely instead on the vasculogenic properties of EPCs and their development in correlation with VSMCs, myoblasts and fibroblasts to obtain a viable network of vessels (Lesman et al., 2011; Alekseeva et al., 2014); however, these networks are rapidly restructured *in vivo*, and substituted with mature vessel which conform to the structure of the implant (Bae et al., 2012; Hanjaya-Putra et al., 2013). This means that these implants will undergo a vascularization process *in vivo* that will largely supersede the one completed in the *in vitro* phase; in the case of more mature vessels, the construct will connect to the existing vasculature *in vivo* through “wrapping and tapping” anastomoses (Cheng et al., 2011); after this passage, the construct will be replace with cells and matrix from the host's tissues, in an inflammation-like process requiring the chemotaxis of progenitors cells (Roh et al., 2010).

### *In vivo* vascularization

The development of blood vessels *in vivo* takes advantage of the host's regenerative capabilities, which can be regulated varying the cellular and chemical composition of the implanted device. The different phases that follows implantation recapitulate, optimally, those observed in regeneration, steering the wound healing process from a fibrotic/scarring outcome to a functional one (de Jonge et al., 2014).

While regenerative processes are necessary for the maturation of the former constructs into functional tissue, the immediate reaction to the engineered device is fundamental for the generation of a viable vessel network, since the *in vivo* vascularization requires a delicate balancing of the different phases of wound healing and regeneration following the implantation, so to achieve the necessary chemotaxis of progenitor cells and paracrine regulators without incurring in a state of prolonged inflammation (Gurtner et al., 2008).

There are two main tools with which to achieve this goal: the cellular component of the device and the structure and functionalization of the scaffold. The cellular components have been selected from autologous (Conconi et al., 2005), syngeneic, allogeneic (Corona et al., 2014) or xenogeneic (Kang et al., 2011) sources, and have been used directly or subsequently to genetic modification intended to increase their effectiveness through augmented expression of growth factors and cytokines (De Coppi et al., 2005). As for the scaffold, its structure can retain chemical signals and modify their local concentration, either geometrically or by surface modification which bind and/or release these factors at a controllable rate; similarly, scaffold derived from the decellularization of tissues are treated to minimize the immune reaction of a xenogeneic host (Badylak, 2014). Moreover, the rate of degradation, and the mechanical and topographical characteristics of the scaffold modify the behavior of the surrounding cells (Zhang et al., 2014), and therefore of the vascular compartment; uniform, randomized geometry, such as interconnected pore networks, facilitate the vascularization process, without imparting a definitive structure of the vascular compartment (Levenberg et al., 2005). However, since a functional design is proved to be advantageous in the integration of SMTE constructs (Koffler et al., 2011), and given that the process of vascularization is inextricably bound to the structural development of living tissues (Tirziu and Simons, 2009; Lesman et al., 2010), it is reasonable to assume that the presence of a defined architecture prior to implantation will inform the structure of the resulting implant, facilitating its integration in tissues characterized by a high degree of anisotropy such as skeletal muscle (Takaza et al., 2013).

## Mechanical and physical stimuli

Cells are sensitive to mechanical and physical changes in their environment, such as the bidimensional or tridimensional structure of the scaffold, its stiffness, or the presence of a voltage across the culture medium; recently, the effect induced by these parameters on cellular behavior have been studied *in vitro*, in the hope to obtain versatile tools for tissue engineering not subjected to the drawbacks suffered by biochemical signals, namely difficult application, limited availability and restrictive regulations.

The cellular mechanisms responsible for the translation of these signals into complex behavior are still being studied, notably in the engineering of tissues with load-bearing and dynamic functions (Guilak et al., 2014). Cells are able to feel and interact with their environment through specialized clusters of transmembrane proteins forming focal adhesions (FAs) (Eyckmans et al., 2011). These structures connect extracellular proteins and other surfaces viable to attachment to the cytoskeleton, the complex system of structural proteins that functions as an actuator of mechanical actions and as a sensor to the environment (Discher et al., 2005), translating its deformation into transcription factors headed for the nucleus; moreover, this structure forms a mechanical connection the nucleus and the Golgi Apparatus to the exterior of the cell (Wang et al., 2009), suggesting a close relationship between the shape of nucleus and the behavior of the cell (Nava et al., 2014).

In particular, the cytoskeletal architecture of the skeletal muscle is intrinsically related to its function and mediates the mechanical stimuli required in the maturation of tissue engineering constructs (Vandenburgh et al., 1988, 1991, 1999). In the original work of Vandenburgh et al. chicken myoblasts seeded on a collagen gel were exposed to stimuli meant to simulate processes observed in embryos, in accordance with the generally agreed principle that regeneration of tissue recapitulates embryonic development (Chargé and Rudnicki, 2004); the protocol, with some variations (Boonen et al., 2010), consists in an initial stretching corresponding to the elongation of the bone, followed by intermittent contractions similar to the impulses derived from postnatal stimuli (Riboldi et al., 2008). Skeletal muscle cells exposed to the correct stimuli show increased expression of differentiation markers compared to static cultures (Candiani et al., 2010), and progress into the formation of polynucleate syncytia; the reaction to cyclic strain, however, depends heavily on the amplitude and frequency of the stimuli: the same line of myoblasts have shown different reactions to alternate protocol and, interestingly, to alternate topographies. It has been shown (Ahmed et al., 2010) that myoblast cell lines tend to align to grooves in the substrate, but they respond to cyclic tension by forming striates stress fibers angled at 45° with respect to the direction of the deformation; finally, on flat surfaces, cyclic mechanical stretching induces fiber angled ad 70° to the stretching axis, attributed to the perpendicular contraction due to the Poisson's ratio of the material. Given the complex behavior exhibited by the cells in response to mechanical stimuli, a reliable protocol for cellular alignment would prove of great value in the engineering of tissues with specific geometric requirements (Li et al., 2013b).

An interesting interpretation has been proposed recently Equation (1) (Livne et al., 2014), which considers the dissipation of cellular elastic energy as the principal reorientation factor The resulting model predicts the orientation angle of the cells θ based on a dimensionless parameter specific of the cell line and the ratio between principal strain and the Poisson ratio of the support:

(1) $$\bar{\theta }=\text{arcos}\left(\sqrt{b+\frac{1-2b}{r+1}}\right)=\text{arctan}\left(\sqrt{\frac{r+b\;\cdot \;\left(1-r\right)}{1-b\;\cdot \;(1-r)}}\right)$$

where r is the negative inverse of the ratio between the principal strain and its perpendicular counterpart, and b is a dimensionless parameter that depends from the ratio between the cellular Young modulus along its main orientation axis (E_θθ_) and that along the perpendicular axis (E_ρρ_). Although this model is capable of predicting the orientation angle well within a significant confidence interval in a bidimensional setting, it is yet to be tested with multiple cell lines or in a three dimensional environment, a setting that is known to influence cell behavior in a complex manner (Boonen et al., 2010). Nevertheless, it provides a relatively straightforward interpretative key to the disposition of cells in load bearing and dynamic tissues, provided of course that the parameter *b* could be used in cells with high aspect ratio such as myocytes.

While the disposition of the cells in response to mechanical stimuli can be approximated to a purely physical process, growth, differentiation and function are far more complex behaviors; a viable avenue for this kind of research lies in the automated analysis of the cellular response to every possible combination of multiple factors. This technique can provide a unifying research framework and it can be used to rapidly individuate unexpected cellular responses to specific sets of stimuli. Similar approaches have been used to determine the reactions of undifferentiated cells to 2D topography (Unadkat et al., 2011), biomaterial composition (Mei et al., 2010), stiffness in 3d scaffolds (Sala et al., 2011) and a combination of 3D scaffolds and growth factors (Ranga et al., 2014). On a final note, it is worthwhile to mention that these combinatorial studies are also limited in that they expose the cell to repetitive stimuli that do not accurately replicate the environment: the ECM is characterized by a controlled randomness, which has proven to influence cell behavior in bidimensional studies (Dalby et al., 2006; Biggs et al., 2010); it can be argued then that these studies can only provide a framework for the identification of complex stimuli to be analyzed subsequently with more accurate techniques.

## Clinical applications—present and future

As of now, SMTE constructs are either intended as *in vitro* skeletal muscle disease models (Gayraud-Morel et al., 2009; Vandenburgh, 2010; Kelc et al., 2013) (Table 1) or as *in vivo* preclinical research tools (Turner et al., 2012; Carosio et al., 2013; Mertens et al., 2014) (Table 2).

**Table 2:** ***In vivo* models of interest**.

**References**	**Model**	**Scaffold**	**Construct type**	**Relevant details**
Corona et al., 2014	Heterologous MDCs	BAM	TA VML; Lewis rats	Uniaxial mechanical strain
Juhas et al., 2014	GCaMP3+ Sprague-Dawley muscle tissue	Fibrin/Matrigel	*In vitro* model; dorsal skinfold window chamber in nude mice	Self contraction; *in vitro* maturation; *in vivo* vascularization; *in vivo* CTX damage regeneration
Carosio et al., 2013	Autologous muscle tissue	Autologous ECM	EDL VML; C57BL6 WT, MLC/hAP	Self assembly, self contraction; response to electrical stimuli; vascularization
Corona et al., 2013a	Autologous muscle tissue	Autologous ECM	TA VML; Lewis rats	*In vivo* filler
Criswell et al., 2013	GFP-FVB MPCs; HUVECs; 10T1/2 cells	Matrigel	Subcutaneous insertion in nude mice	*In vivo* activity of ECs and pericytes on SMTE constructs
Haraguchi et al., 2012	Rat cadiac cells; HUVECs; HSMMCs	PNIPAAm substrate; fibrin, gelatin substrate; self produced ECM	Subcutaneous dorsal insertion, transplantation onto infarcted heart in F344 nude mice	Cell sheet stacking; electrical stimuli maturation; *in vitro* vascularization
Williams et al., 2012	Explanted soleus muscle cells	Autologous ECM on fibrin	Implantation along the VL, near the sciatic nerve. Innervation with sural nerve in F344	Self contraction and effect of innervation with host's nerve; shift in myosin type
Koffler et al., 2011	C2C12; HUVECs; human foreskin fibroblasts	Surgisis SIS	Full thickness abdominal wall replacement in nude mice	Cocolture; variable *in vitro* conditioning period
Levenberg et al., 2005	C2C12; HUVECs; mouse EFs	PLLA-PGA porous scaffold	Dorsal midline subcutaneous implantation in CB17 SCID	Coculture; prevascularization
Sicari et al., 2014	–	SIS	ATC (3), quadriceps (2) VML	Clinical trial
Mase et al., 2010	–	BAM	Quadriceps femori VML	Clinical case

*BAM, Bladder Acellular Matrix; CTX, CardioToXin; EDL, Extensor Digitorum Longus; EF, Embryonic Fibroblasts; HSMMC, Human Skeletal Muscle Myoblast Cells; HUVEC, Human Umbilical Vascular Endothelial Cell; MPC, Muscle Progenitor cell; PGA, PolyGlicolic Acid; PLLA, Poly-L-Lactic Acid; PNIPAAM, poly-N-IsoPropylAcrylAmide; SIS, Small Intestine Submucosa; TA, Tibialis Anterior*.

The only regenerative medicine devices to have reached clinical application are acellular compounds derived from animal ECM used *in loco* to improve the motility and strength of skeletal muscles subjected to traumatic injury (Mase et al., 2010; Sicari et al., 2014), or cell therapy strategies that are injected locally and systematically to improve syndromes stemming from genetic diseases (Tedesco et al., 2010; Tedesco and Cossu, 2012). While these therapies use tissue engineering techniques, they do not fall into the classic definition of TE constructs (Langer and Vacanti, 1993), since they lack either a cellular component or a scaffold and are not subjected to maturation in a bioreactor. Nonetheless, it is likely that future TE devices will refer to these forerunners as a model on which to improve, and that further early approaches in this field will implement methodologies that have proven most effective on their own in clinical trials.

### Toward a human host

In the translation to the clinic, TE devices face a unique set of challenges due to their being artificial constructs made of synthetic and biologic material, the latter being of autologous, syngeneic or even xenogeneic origin (Mertens et al., 2014); setting aside the regulatory aspects of this progression, which have been recently described in details (Pashuck and Stevens, 2012; Fisher and Mauck, 2013; Martin et al., 2014) there are some aspects in the development of a preclinical engineered skeletal muscle device that are of particular relevance when considering clinical applications.

Most of the SMTE constructs that are currently being developed rely on small mammals as animal models to study their effects on muscle repair (Table 2); these models are reliable, easily available and provide comparable result between different laboratories, yet they also suffer from substantial limitations in the translation to the clinic (Boldrin et al., 2010; Seok et al., 2013; Bareja et al., 2014). In most cases, human cell lines and primary cells are used to develop constructs that are subsequently implanted in athymic models, lacking therefore the ability to mount an adaptive immune response; these models allow for the maturation of the implant in a living subject, under the reasoning that subsequent human trials would rely on autologous cells and scaffolds proven to be clinically compatible. However, the reaction to the human molecular apparatus found in the cellular compartment of the devices may differ from that originating from the host (Borisov, 1999). The predictive capability of these models in regard to cellular behavior under growth factor stimulation was put into question in a recent paper (Mujagic et al., 2013), where the authors suggested that the effects of different species' isoforms of the growth factor VEGF vary greatly depending on the recipient cells: this would explain the apparent inability of human VEGF to cause angiomas in mouse models, which could lead to an underestimation of the side effects of modified cells in autotransplantation cases. As a consequence, other studies which propose the use of genetically modified cells to improve the vascularization of regenerating muscles (Gianni-Barrera et al., 2013; Shevchenko et al., 2013) may have to compensate the effects observed in animals as to avoid the formation of irregular vasculature in human hosts; moreover, these uncertainties are also observed in clinical cases involving novel combinations of accepted materials and cellular populations, particularly undifferentiated staminal cells, which, if unregulated, could lead to ectopic tissue formation and neoplastic lesions (Amariglio et al., 2009; Thirabanjasak et al., 2010).

There are currently few ways to solve this problem, as the understanding of the interaction between host and implant is still largely incomplete, particularly in regard to the role of the immune system (Bohgaki et al., 2007); however, given the urgent need for reliable constructs capable of supplementing and replacing tissue transplants, alternative solutions to this problem could be found in therapies that do away entirely with the cellular component of the graft (Burdick et al., 2013), or in the development of cellular populations such as autologous induced pluripotent stem cells (iPSCs) (Takahashi and Yamanaka, 2006), which could solve the problems correlated with allotransplantation (Araki et al., 2013). Nevertheless, as these cells are capable of forming teratomas (Knoeplfer, 2009), their usage in clinical setting is problematic; protocols intended to produce differentiated cells from iPSCs have proven effective in limiting the occurrence of tumors both in mice (Liu et al., 2013; Suzuki et al., 2013) and in non-primate models that more closely resemble human physiology (Hong et al., 2014); as this line of research progresses, it will be feasible to use this technology to develop constructs from the patient's own differentiated cells, in a clinical testing framework offsetting potential benefits from limited risks (Goldring et al., 2011).

Another topic that will need to be addressed before the clinical application of SMTE constructs is also correlated to the immune reaction to the devices and to the conditions present at the site of implantation: these devices are *in situ* implants in recently wounded sites which do not replicate the conditions of VML encountered in clinical setting (Grogan et al., 2011; Li et al., 2013a): it is not common practice to develop animal models of VML in which the site of implantation had already undergone a normal repair process which resulted in the formation of scar tissue. As the homeostasis of the site of injury is much different compared to that of uninjured tissues, the predictive value of the animal models is diminished, and even more so when the damage is compounded by genetic mutations leading to defects in the repair process (Bosurgi et al., 2011).

### Current approaches

There are numerous hurdles to be overcome before SMTE constructs will have a use in clinical settings; to the best of our knowledge, there have only been two clinical applications of regenerative medicine devices for VML in skeletal muscle, both involving *in situ* implantation of decellularized porcine small intestinal submucosa (SIS). In the first instance, a clinical case, a large volumetric defect in the quadriceps femori, treated 3 years before with a latissimus dorsi muscle flap (Mase et al., 2010), was treated with an acellular SIS scaffold. Following the positive outcome of this experiment a clinical trial involving five patients and a different scaffold (porcine bladder matrix) was recently published (Sicari et al., 2014), showing that the treatment did not induce an adverse effect [as xenogeneic ECM would if not completely decellularized (Keane et al., 2012)] and improvement in strength and mobility in three cases, compared to a patient-specific baseline. While this device cannot be considered the result of tissue engineering according to the usually accepted definition (Langer and Vacanti, 1993), it provides evidence that a commonly used tissue engineering scaffold can improve skeletal muscle injuries; moreover, acellular scaffolds such as SIS have shown performance comparable to decellularized muscle tissue in SMTE treating of VML (De Coppi et al., 2006; Wolf et al., 2012), which could obviate the need for human tissue as a starting material for scaffold development.

These results, and other reports analyzing the effect of empty scaffolds on cell homing (Ju et al., 2014), suggest that SMTE materials may indeed constitute a valid clinical options for the treatment of VML. A better understanding of the role of the cellular component of the devices will be required, as evidence in other engineered tissues have proven a limited structural role for the seeded cells, which are rapidly overcome and substituted by the host's own (Hibino et al., 2011).

### Future directions

Tissue engineering and regenerative medicine treatment of skeletal muscle VML is intrinsically dependent on the dimensions of the devices meant to substitute the missing tissues: the lesions to be treated are extensive by their very own nature, and require thick flaps that are beyond what is currently possible to reproduce through TE constructs. Given the unique characteristics of skeletal muscle tissue, clinically relevant SMTE constructs will require techniques aimed at the development of functional structures of large scale in the three dimensions. The techniques that are going to see clinical application in the most immediate future are those which are derived from accepted clinical methodologies, which allow the introduction of fewer variables to be tested in the preclinical phases.

In this respect, decellularized acellular devices possess many characteristics that make them suitable for a rapid translation to a clinical application: the ECM derived from large mammals has seen ample use as grafts in vascular settings (Breymann et al., 2009), and the structure and composition of the decellularized material is capable of promoting the vascularization of the structure as it is assimilated in the body (Burdick et al., 2013; Teodori et al., 2014); nonetheless, concerns about the complete decellularization of the materials are still present, as residual traces of cellular material can lead to the failure of the device (Keane et al., 2012). An alternative to the use of heterologous material is the production of *in vitro* ECM from cell cultures derived from the host: this approach allows for less stringent decellularization parameters, and provide a molecular milieu closely resembling that of the original tissue, lacking however its highly organized architecture, as these devices are produced with synthetic scaffolds (Lu et al., 2011) or directly from connected cell sheets (Dahl et al., 2011). For this methodology to be applicable in VML treatment, the *in vitro* maturation should produce either a single construct with patent vessels (Gualandi et al., 2013) or multiple layers complete of vascular structure which would anastomose when placed in close contact. The latter approach has met with some results in recent reports (Sakaguchi et al., 2013; Sekine et al., 2013), where multiple cell sheets have been stacked to achieve multiple layer thickness to produce cellular constructs that were directly implanted in animal models. While the constructs obtained through this methodology are still far from the requirements of skeletal muscle VML treatments, they have been proven advantageous in the treatment of a case of dilated cardiomyopathy (DCM) (Sawa et al., 2012); although the dimensions of the implants (40 mm of diameter and a thickness of four cell layers) were scarcely comparable to those required for VML, this case constitutes an important precedent for the application of cell sheet engineering in the treatment of striated muscle disorders.

Therefore, it is foreseeable that clinical SMTE will proceed gradually incrementing the complexity of the implanted devices, giving preference to methodologies that implement cellular populations subjected to minimal *in vitro* treatment, or none at all.

## Conclusions

SMTE has seen a rapid development in the last decade, facilitated by the growing understanding of the processes underlying the regeneration of this tissue and the concomitant formation of vascular networks. The latter topic is of particular interest for the production of clinically relevant constructs, as the treatment of VMLs requires large, mature engineered tissues with stable vessel networks. The structure of the scaffold and mechanical stimuli applied during the *in vitro* phase of development have emerged as viable tools for the production of ordered, vascularized tissues with significant prospective clinical applications. As decellularized materials are undergoing clinical trials, it is foreseeable that clinical devices composed of ordered scaffolds and autologous vascular compartments will also be used in the treatment of VMLs.

### Conflict of interest statement

The authors declare that the research was conducted in the absence of any commercial or financial relationships that could be construed as a potential conflict of interest.
